# Identification and genetic characterization of two conjugative plasmids that confer azithromycin resistance in *Salmonella*

**DOI:** 10.1080/22221751.2022.2058420

**Published:** 2022-04-11

**Authors:** Miaomiao Xie, Kaichao Chen, Edward Wai-chi Chan, Sheng Chen

**Affiliations:** a Department of Infectious Diseases and Public Health, Jockey Club College of Veterinary Medicine and Life Sciences, City University of Hong Kong, Kowloon, Hong Kong; b State Key Lab of Chemical Biology and Drug Discovery, Department of Applied Biology and Chemical Technology, The Hong Kong Polytechnic University, Hung Hom, Hong Kong

**Keywords:** *Salmonella*, conjugative plasmid, azithromycin resistance, virulence plasmid, evolution

## Abstract

With the development of multidrug resistance in *Salmonella* spp. in recent years, ciprofloxacin, ceftriaxone, and azithromycin have become the principal antimicrobial agents used for the treatment of *Salmonella* infections. The underlying mechanisms of plasmid-mediated ciprofloxacin and ceftriaxone resistance have attracted extensive research interest, but not much is focused on azithromycin resistance in *Salmonella*. In this study, we investigated the genetic features of two conjugative plasmids and a non-transferable virulence plasmid that encode azithromycin resistance in food-borne *Salmonella* strains. We showed that the azithromycin resistance phenotype of these strains was conferred by *erm(B)* gene and/or the complete genetic structure IS*26-mph(A)-mrx-mphR-*IS*6100.* Comparative genetic analysis showed that these conjugative plasmids might originate from *Escherichia coli* and play a role in the rapid dissemination of azithromycin resistance in *Salmonella*. These conjugative plasmids may also serve as a reservoir of antimicrobial resistance (AMR) genes in *Salmonella* in which these AMR genes may be acquired by the virulence plasmids of *Salmonella* via genetic transposition events. Importantly, the formation of a novel macrolide-resistance and virulence-encoding plasmid, namely pS1380-118 kb, was observed in this study. This plasmid was found to exhibit transmission potential and pose a serious health threat as the extensive transmission of azithromycin resistant and virulent *Salmonella* strains would further compromise the effectiveness of treatment for salmonellosis. Further surveillance and research on the dissemination and evolution routes of pS1380-118kb-like plasmids in potential human pathogens of the family of *Enterobacteriaceae* are necessary.

## Introduction

*Salmonella* is a primary cause of food-borne diseases and is considered to be a major public health threat worldwide [1,2]. Most cases of salmonellosis are self-limiting, yet antimicrobial treatment is necessary in cases of systematic and serious infection, especially in immune-compromised and elderly patients [3]. Due to the increasing incidence of resistance to ciprofloxacin and ceftriaxone, azithromycin is regarded as a last sort, FDA-approved antimicrobial agent for the treatment of systemic *Salmonella* infections, especially those caused by *S. Typhimurium* [4]. Azithromycin is a semisynthetic macrolide antibiotic derived from erythromycin by methyl substitution of a nitrogen atom in the macrolide ring. It differs structurally from erythromycin and exhibits stability in an acidic environment, a wide antibacterial spectrum, negligible irritability, and rapid absorption from gastrointestinal (GI) tract upon oral administration [5], and has become one of the most commonly used antimicrobials after being launched in 1991 [6]. The excellent pharmacological characteristics and favourable membrane permeability of azithromycin allow it to be used for the treatment of infections caused by various members of *Enterobacteriaceae*, especially these common foodborne pathogens of *E. coli*, *Shigella* spp., or *Salmonella* spp. [7,8]. The key antimicrobial mechanism of azithromycin involves inhibition of protein synthesis and bacterial growth by binding to the bacterial ribosome and therefore hindering mRNA translation. To date, two other mechanisms of azithromycin resistance have been reported: one is associated with extruding antibiotics from bacterial cytoplasm by efflux. Relevant bacterial efflux system families are ABC, MFS, MATE, RND, and SMR, among which genes encoding the ABC family efflux pumps including *msr(A)*, *msr(D),* and *msr(E)*, and those which encode the MFS family, such as the *mef(A)* and *mef(B)* genes, are transferable among different bacterial pathogens [9,10]. The second mechanism involves the modification of either the bacterial ribosome or the molecular structure of macrolides by rRNA methylases, esterases, and phosphorylases, which are encoded by the *erm*, *ere(A)*/*ere(B)* and *mph(A)*/*mph(B)* genes, respectively [11]. However, plasmids carrying these determinants mediating azithromycin resistance are sporadically reported in *Salmonella* strains, among which plasmids of IncHI2 and IncC types were reported in recent years [12–14], and most of these plasmids originate from other *Enterobacteriaceae*. In addition, extensively drug resistant (XDR) *S. typhimurium* with a single point mutation in the efflux pump AcrB (R717Q/L) is the main epidemic azithromycin resistant isolate in recent years in Southeast Asian countries, especially in Nepal, India, and Pakistan [15].

In this study, we performed genetic characterization of two conjugative plasmids that carry an rRNA methylase gene *erm(B)* and a phosphorylase-encoding cluster with the structure IS*26-mph(A)-mrx-mphR-*IS*6100.* One was an IncI1 type plasmid that can acquire macrolide-resistance genes and was responsible for causing an increasing prevalence of azithromycin resistance in *Salmonella*. The other was an IncFIC/IncFIB type plasmid, which was commonly detected in *E. coli* strains and responsible for transferring azithromycin resistance genes to a typical virulence plasmid, forming a novel azithromycin resistance and virulence-encoding plasmid in *S. typhimurium*. These events would lead to an increase in the incidence of life-threatening *Salmonella* infections and pose a serious threat to human health.

## Materials and methods

### Bacterial isolation and antimicrobial susceptibility tests

*Salmonella* strains S1330 and S1380 were isolated from retail pork samples collected from supermarkets in Shenzhen, China in 2013. Species identification was performed by detection of the *Salmonella*-specific *invA* gene and by using the MALDI-TOF-MS Biotyper System (Bruker, Germany). The serotypes of the two strains were then identified according to the Kauffmann-White scheme, using a commercial antiserum (Difco, Detroit, MI). Antimicrobial susceptibility tests were performed by following the microdilution method recommended by Clinical and Laboratory Standards Institute [16]. *Escherichia coli* strain ATCC 25922 and *Staphylococcus aureus* strain ATCC 29213 were used as quality control.

### Cloning of two resistance determinant-bearing clusters IS*26-mph(A)-mrx-mphR-*IS*6100*, IS*26-mph(A)-mrx-ΔmphR-*IS*26,* and *mph(A)* gene

The corresponding gene fragments were amplified using the primers listed in Table 2. Briefly, sequences of 200 bp located up- and downstream of the target genes, including the natural promoters, were amplified. PCR products were then ligated to the cloning vector pBackZero-T, generating pBackZero-*mph(A)*, pBackZero-IS*26-mph(A)-mrx-mphR-*IS*6100* and pBackZero-IS*26-mph(A)-mrx-_Δ_mphR-*IS*26* respectively, which were then transformed into *E. coli* strain DH5α. Transformants were selected on LB plates containing 50μg mL^−1^ kanamycin, followed by confirmation of their genetic identity through PCR screening with the cloning primers described above.

### Conjugation and pulsed-field gel electrophoresis (PFGE) analysis

Conjugation experiments were performed to evaluate the transmission potential of azithromycin resistance in *Salmonella* strains, with a sodium azide resistant *E. coli* strain J53 being used as the recipient. In brief, overnight culture of the test *Salmonella* strains and the recipient were diluted 100-fold in fresh Luria Bertani (LB) broth and incubated at 37°C for 4 h until the logarithmic phase was reached. The *Salmonella* strains and the recipient were mixed at a ratio of 1:4 and inoculated onto a 0.45 µm membrane placed on LB agar. After incubation for 24 h at 37°C, serial dilutions of the bacterial mixture were incubated onto Eosin Methylene Blue (EMB) agar supplemented with 100 µg mL^−1^ sodium azide and 16 µg mL^−1^ azithromycin for selection of transconjugants. The transconjugants were then identified by antimicrobial susceptibility tests and pulsed-field gel electrophoresis (PFGE) as previously described [17].

### Plasmid sequencing and bioinformatics analysis

Plasmids harboured by *Salmonella* strains S1330 and S1380 were extracted using the Qiagen Plasmid Midi Kit (Qiagen, Valencia, CA) and then sequenced by the NextSeq Illumina platform (San Giego, CA) and Nanopore MinION (long-read) sequencing platform, using the MinION R9.4.1 flow cell [18]. The complete plasmid sequence was acquired by assembling the short and long reads obtained from both Illumina and Nanopore platforms by Unicycler v0.4.9b [19], and then annotated by Rapid Annotation using Subsystem Technology (RAST) version 2.0 [20]. Antimicrobial resistance genes, insertion sequences and plasmid incompatibility types were identified by ResFinder [21], ISFinder [22], and PlasmidFinder [23] tools. Sequence comparison of plasmids was conducted using BLAST Ring Image Generator (BRIG) [24] and Easyfig [25].

## Results

### Phenotypic characterization of *Salmonella* strains

**Figure 1.**
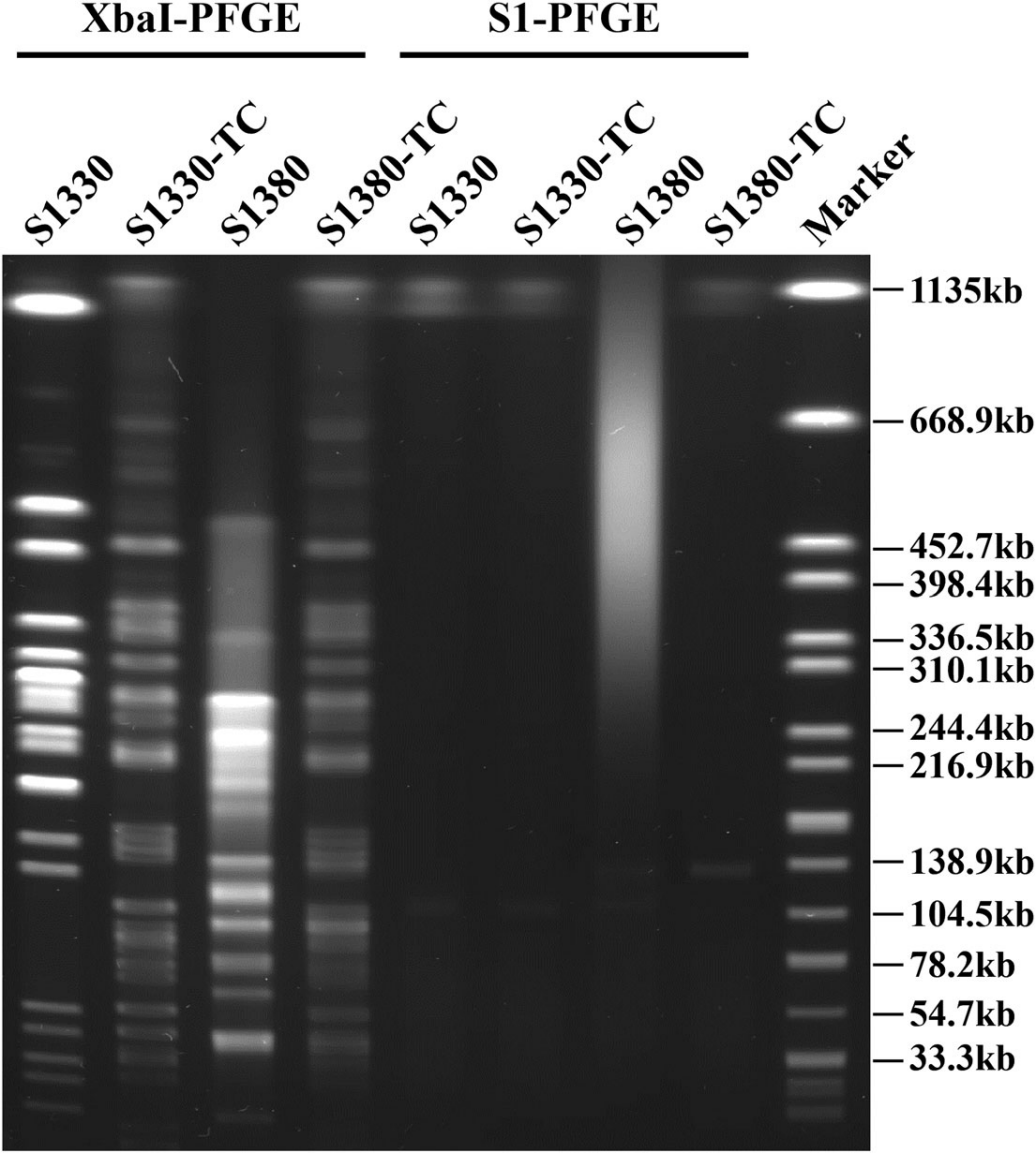
**PFGE profiles of *Salmonella* strains S1330 and S1380 and the corresponding transconjugants.** XbaI-PFGE and S1-PFGE profiles of *Salmonella* strains S1330, S1380 and their transconjugants are shown.

*Salmonella Derby* strain S1330 and *Salmonella Typhimurium* strain S1380 were isolated from retail pork samples in Shenzhen and found to be resistant to most of the antibiotics tested, including azithromycin, chloramphenicol, ciprofloxacin, kanamycin, nalidixic acid, trimethoprim/sulfamethoxazole, and tetracycline, but the strains remained susceptible to amikacin and meropenem (Table 1). Conjugation assays indicated that the azithromycin resistance phenotype of both isolates could be transferred to the recipient *E. coli* J53 upon selection by azithromycin. The transconjugants S1330-TC and S1380-TC exhibited dramatic increase in minimum inhibitory concentration (MIC) of azithromycin when compared with the recipient *E. coli* J53, with more than 128-fold increase in azithromycin MIC being observed (Table 1). S1 nuclease pulsed-field gel electrophoresis (S1-PFGE) illustrated that both the *S. Derby* strain S1330 and the corresponding transconjugant contained a single plasmid of ∼110 kb, which was designated as plasmid pS1330-110 kb. In addition, S1-PFGE results showed that *S. Typhimurium* strain S1380 harbored two plasmids, the size of which were found to be ∼118 and ∼135 kb by plasmid sequencing. However, only the larger plasmid (∼135 kb) could be transferred to the recipient by conjugation (Figure 1). These two plasmids were designated as plasmid pS1380-118 kb and pS1380-135 kb, respectively.

**Table 1.** Phenotypic characteristics of Salmonella strains tested in this study.

Strain	Species	Source	MIC (µg mL^−1^)										
			AMK	AMP	AZI	CHL	CIP	CTX	KAN	MRP	NAL	SXT	TET
S1330	*S. Derby*	Pork	8	> 64	> 128	> 64	4	0.25	> 128	0.03	64	> 32	> 32
S1330-TC	*E. coli*	/	8	> 64	> 128	1	≦ 0.06	≦ 0.06	16	0.03	2	4	0.5
S1380	*S. Typhimurium*	Pork	4	> 64	> 128	> 64	64	> 128	> 128	0.03	> 64	> 32	> 32
S1380-TC	*E. coli*	/	2	> 64	> 128	1	≦ 0.06	≦ 0.06	> 128	0.03	2	4	0.5
J53	*E. coli*	/	≦ 0.5	2	1	1	0.015	≦ 0.015	≦ 0.5	0.03	2	4	0.5

AMK, amikacin; AMP, ampicillin; AZI, azithromycin; CHL, chloramphenicol; CIP, ciprofloxacin; CTX, cefotaxime; KAN, kanamycin; MRP, meropenem; NAL, nalidixic acid; SXT, trimethoprim/sulfamethoxazole; TET, tetracycline.

### Genetic analysis of plasmids that confer phenotypic resistance to azithromycin in *Salmonella*

**Figure 2.**
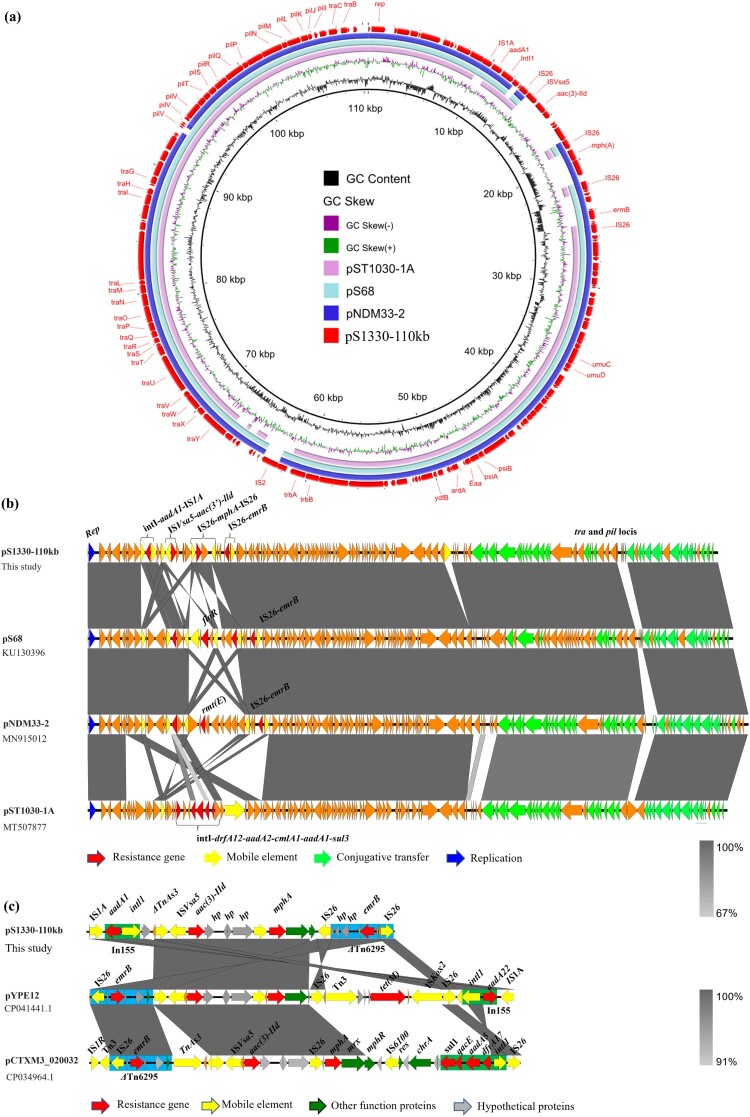
**Genetic structure of plasmids that confer azithromycin resistance in *Salmonella* strain S1330.** Circular (a) and liner (b) alignment of plasmid pS1330-110 kb in *S. Derby* strain S1330 with plasmids deposited in the NCBI database including pNDM33-2 (MN915012), pS86 (KU130396), and pST1030-1A (MT507877) using Blast Ring Image Generator (BRIG) and EasyFig. (c) Liner alignment of the MDR region of plasmid pS1330-110 kb with the plasmids pYPE12 and pCTXM3-020032. Dark blue, gene encoding replication initiation protein; red, resistance gene; yellow, insertion sequence; green, gene encoding plasmid conjugative transfer protein Tra and Pil.

Complete sequences of plasmids pS1330-110 kb, pS1380-118 kb and pS1380-135 kb were acquired using both NextSeq Illumina and Nanopore MinION sequencing platforms. Plasmid pS1330-110 kb was 110,440 bp in length, exhibited a GC content of 50.2% and comprised 147 predicted coding sequences (CDS). The plasmid was found to belong to IncI1 type. This plasmid was found to harbour several resistance genes including *mph(A)*, *aadA1*, *aac(3)-IId,* and *erm(B)*, which may confer resistance to azithromycin and kanamycin, respectively. BLASTN analysis showed that plasmid pS1330-110 kb exhibited the highest degree of homology to a 115,776 bp IncI1 plasmid, namely pS68 (Accession: KU130396), with 99.98% identity and 91% coverage (Figure 2a), as well as plasmid pNDM33-2 (Accession: MN915012) and pST1030-1A (Accession: MT507877), both recovered from *Escherichia coli* strains and shared similar plasmid backbones **(**Figure 2b**),** with 99.8% identity and 85–94% coverage. Sequencing analysis showed that the backbones of these plasmids were extremely conserved after subtracting the multidrug resistance (MDR) region, a mosaic region that comprised various resistance genes including *mph(A)* and *erm(B)*. The *mph(A)* cluster in plasmid pS1330-110 kb was incomplete in which the *mphR* in the complete *mph(A)* cluster (IS*26*-*mph(A)*-*mrx*-*mphR*-IS*6100*) was truncated and the followed mobile element IS*6100* was replaced by an IS*26*. We confirmed that deletion of the *mphR* gene would affect the function of the *mph(A)*-bearing cluster and restore azithromycin sensitivity, as *E. coli* strain DH5α which acquired IS*26*-*mph(A)*-*mrx*-*ΔmphR*-IS*26* and *mph(A)* exhibited sensitivity to azithromycin (azithromycin MIC of both strains were 1μg mL^−1^), whereas the azithromycin MIC of *E. coli* DH5α which acquired the complete structure of IS*26*-*mph(A)*-*mrx*-*mphR*-IS*6100* increased to over 128μg mL^−1^ (Table 2). Our data confirmed that *erm(B)* on pS1330-110 kb could mediate azithromycin resistance by cloning experiments, which suggested that *erm(B)* was a key genetic element that encoded resistance to azithromycin in *Salmonella* S1330. The role of *erm(B)* was also consistent with our previous clonal study in which transconjugant *E. coli* DH5α-*erm(B)* exhibited a 128-fold increased MIC to azithromycin [26].

**Table 2.** MICs of DH5α strains that contained mph(A)-bearing fragments of different structures and relevant primers used.

Strain	Primers	Amplicon size	AZI MIC (μg mL^−1^)
DH5α	–	–	1
DH5α-pBackZero	–	–	1
DH5α-*mph(A)*	F 5′-ATGCGTGCACTACGCAAAG-3′	1144bp	
	R 5′- CGAGCGGGCTATATCGAC-3′		1
DH5α-IS*26*-*mph(A)*-*mrx*-*ΔmphR*-IS*26*	F 5′-GTCGGTGGTGATAAACTTATCATC-3′		
	R 5′- CAATGTCTGACGCGAAGATCAG-3′	4302bp	1
DH5α-IS*26*-*mph(A)*-*mrx*-*mphR*-IS*6100*	F 5′-CACCAATCTCGACTATGCTCAATAC-3′		
	R 5′-GTGGAACGAAAACTCACGTTAAG-3′	5084bp	> 128
DH5α-*erm(B)*	F 5′-AGAAGGAGGGATTCGTCATG-3′		
	R 5′-TCTTGCTAGTCTAGGGACCT-3′	1138bp	> 128

**Figure 3.**
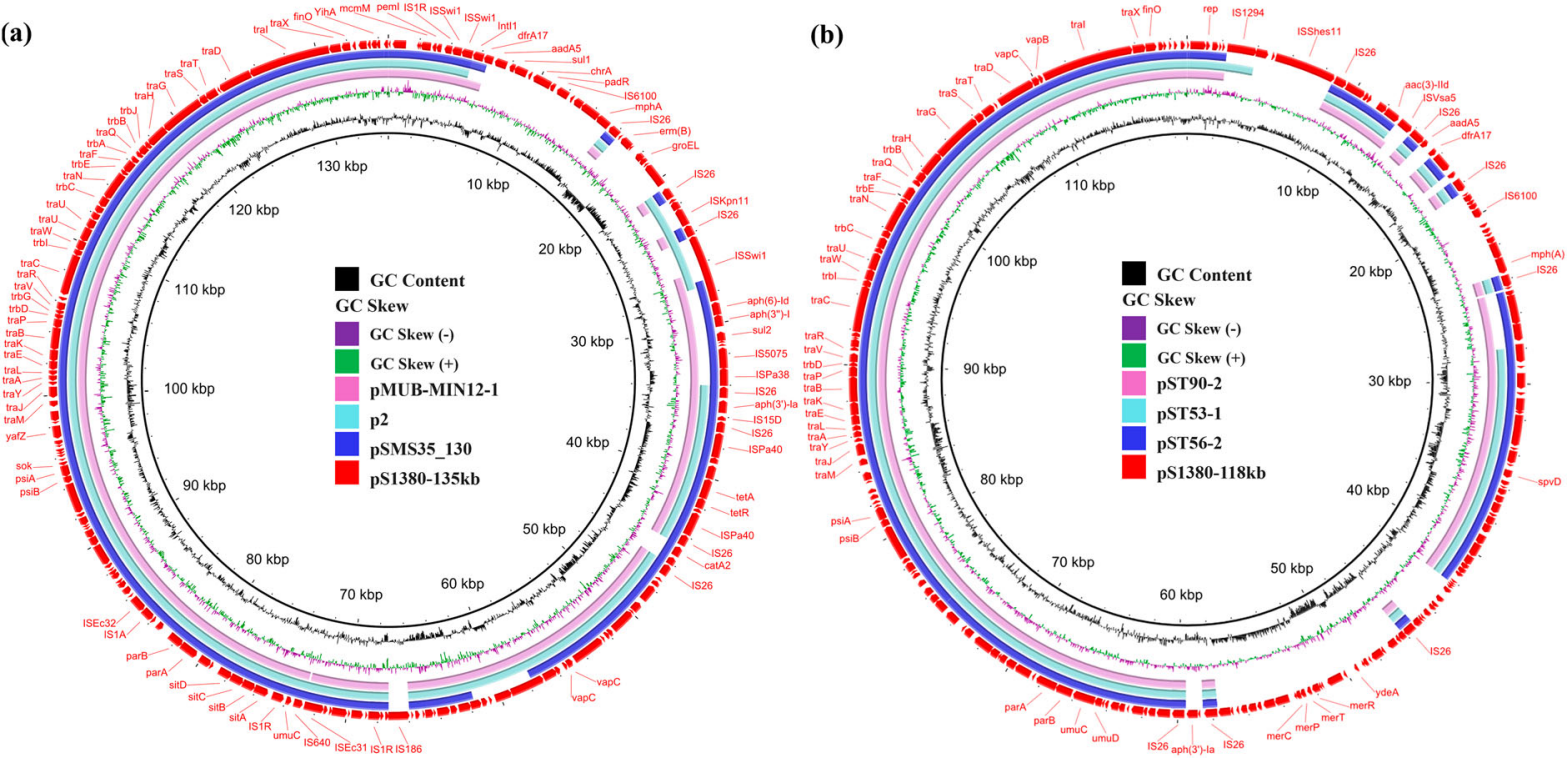
**Genetic structure of plasmids that confer azithromycin resistance in *Salmonella* strain S1380.** (a) Circular alignment of plasmid pS1380-118 kb in *S. typhimurium* strain S1380 with plasmids deposited in NCBI database including pST56-2 (CP050741), pST53-1 (CP050746), and pST90-2 (CP050736), using Blast Ring Image Generator (BRIG). (b) Circular alignment of plasmid and pS1380-135 kb in *S. Typhimurium* strain S1380 with plasmids deposited in NCBI database including pSMS35_130 (CP000971), p2 (LR890271), and pMUB-MIN12-1 (CP069658), using Blast Ring Image Generator (BRIG).

**Figure 4.**
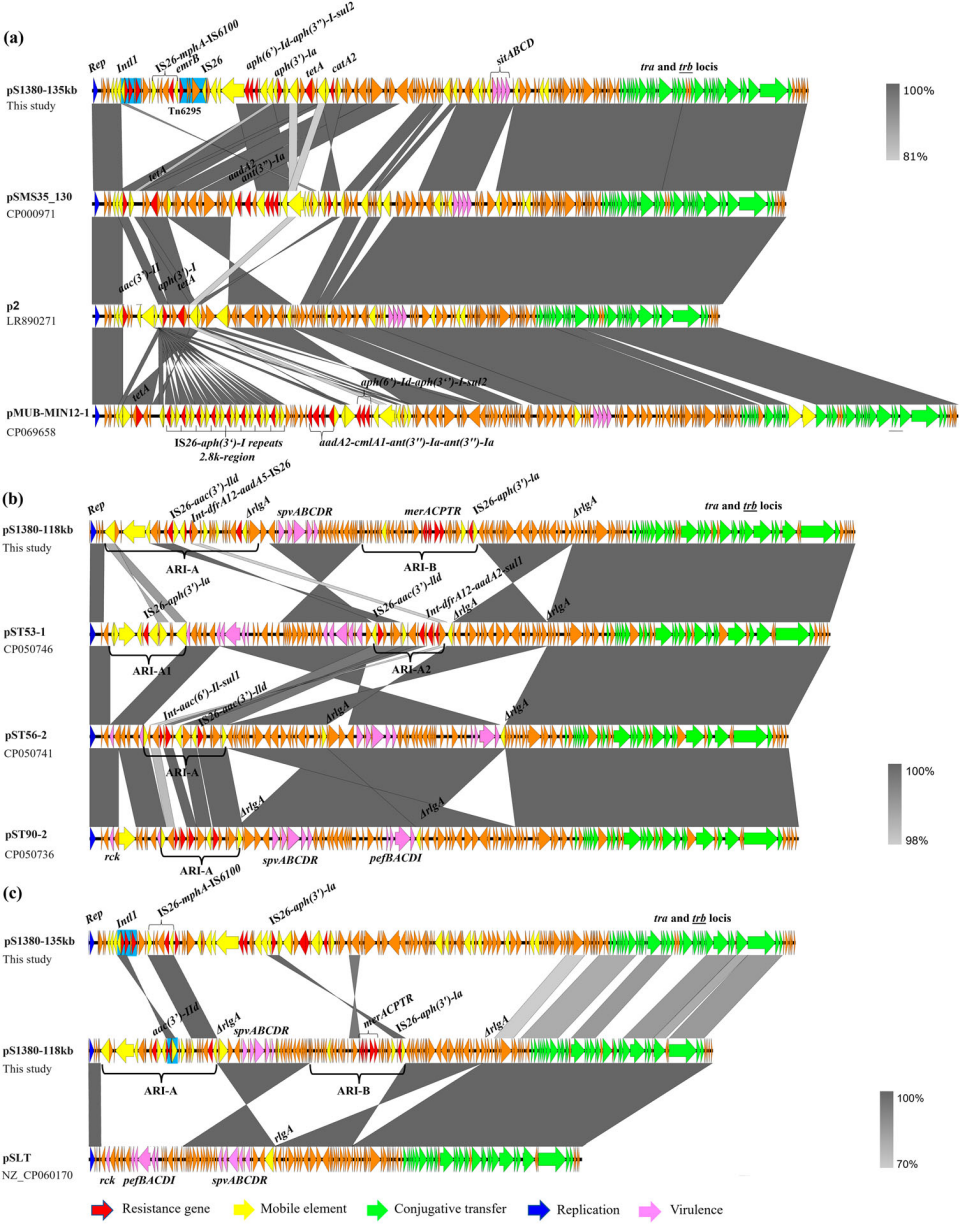
**Genetic structure of plasmids that confer azithromycin resistance in *Salmonella* strain S1380.** Liner alignment of plasmids pS1380-135 kb (a) and pS1380-118 kb (b) with plasmids deposited in NCBI database using Easyfig. (c) Alignment of plasmid pS1380-118 kb with plasmid pS1380-135 kb and virulence plasmid pSLT (CP060170). Dark blue, gene encoding replication initiation protein; red, resistance gene; pink, virulence gene; yellow, insertion sequence; green, genes encoding the plasmid conjugative transfer proteins Tra and Pil.

Plasmid pS1380-135 kb was 134,634 bp in length, with a GC content of 52.1%. The plasmid comprised 184 predicted coding sequences and was found to belong to a IncFIC/IncFIB type plasmid. BLASTN screening against the resistance gene database showed that plasmid pS1380-135 kb harbored multidrug resistance genes, including the aminoglycoside resistance genes *aph(3’’)-I*, *aph(3’)-Ia*, *aph(6’)-Id*, *aadA5* and *acc(3)-II*, the phenicol resistance gene *catA2*, the trimethoprim resistance gene *dfrA17*, the tetracycline resistance gene *tet(A)*, the sulfonamide resistance genes *sul1* and *sul2*, and the macrolide resistance genes *mph(A)* and *erm(B)* (Figure 3a). These resistant determinants located in a ∼40 kb MDR region surrounded by various IS elements with the genetic environments of int-*dfrA17*-*aadA5*-*sul1*, IS*26*-*mph(A)*-*mrx*-*mphR*-IS*6100*, IS*26-erm(B)-groEL-*IS*26*, *Tn3*-*aph(6’)-Id*-*aph(3’)-I*-*sul2*-IS*5075*, and IS*26-aph(3’)-la-_Δ_*IS*26-*IS*26*, respectively. Plasmid pS1380-135 kb exhibited the highest degree of sequence homology (83% overage and 99.87% identity) to a 163,427 bp IncFIC/IncFIB plasmid pMUB-MIN12-1 (Accession: CP069658) recovered from a clinical *E. coli* strain collected from the wound of a patient. The IncFIC/IncFIB plasmid pSMS35_130 (Accession: CP000971) and plasmid p2 (Accession: LR890271), both recovered from *E. coli*, also exhibited similar sequence coverage and identity (Figure 4a). The *aph* and *tet(A)* genes were found in all the IncFIC/IncFIB plasmids, even though a high degree of structural divergence was observed in the mosaic resistance regions of these plasmids. Importantly, the backbone sequences of these plasmids shared a common virulence gene cluster, *sitABCD*, indicating that such plasmids might have originated from pathogenic avian *Escherichia coli* (APEC) strains, as *sitABCD* is commonly located in large conjugative plasmids in APEC strains.

Plasmid pS1380-118 kb was 118,931 bp in length, exhibited a GC content of 53.9% and comprised 190 predicted coding sequences. This plasmid was found to belong to the IncFII type. BLASTN analysis demonstrated that plasmid pS1380-118 kb exhibited homology to various plasmids originated from *Salmonella Typhimurium*, including pST56-2 (Accession: CP050741), with 100.00% identity and 75% coverage, plasmid pST53-1 (Accession: CP050746) with 99.98% identity and 73% coverage, and plasmid pST90-2 (Accession: CP050736) with 99.95% identity and 75% coverage **(**Figure 3b and 4b). In addition, two antimicrobial resistance islands (ARI) (designated as ARI-A and ARI-B) conferring resistance to aminoglycoside and azithromycin were detected in plasmid pS1380-118 kb. Blast against the ResFinder [21] and ISfinder [22] showed that the genetic structure of ARI-A was *int*-*dfrA17*-*aadA5*, IS*Vas5*-*aac(3’)-IId*, followed by a macrolide resistance cluster IS*26*-*mph(A)*-*mrx*-*mphR*-IS*6100*, whereas ARI-B contained a mercury resistance cluster *merACPTR* and an aminoglycoside resistance-encoding transposon with a structure of IS*26*-*aph(3’)-la*­-IS*26* (Figure 4c). The ∼17 kb ARI-B fragment only exhibited high level homology (99.34% similarity, 94% coverage) to plasmid pII (Accession number: LT795504), indicating that the ARI-B region of plasmid pS1380-118 kb might originate from *E. coli* strains such as KV7 [27]. In addition, the plasmid backbone exhibited a high degree of sequence homology to a virulence plasmid, pSLT (Accession number: NZ_CP060170), after removing both ARI regions. Both plasmids contained the virulence locus *spvABCDR*, which encodes important virulence factors reported to play a role in enhancing the growth rate of *Salmonella* strains in mouse model during the systemic phase of infection [28]. Compared with the virulence plasmid pSLT, the ARI-A region in plasmid pS1380-118 kb was found to be replaced by a ∼14 kb region containing the original virulence cluster *pefBACDI* and a virulence gene *rck* **(**Figure 4c**)**. The *pef* operon is associated with fimbriae biosynthesis and contributes to adherence to the intestinal epithelium, whereas the *rck* gene is associated with resistance to complement killing [29]. The formation of ARI-B in plasmid pS1330-118 kb might be associated with the *rlgA* gene, which exhibited high sequence homology to several previously reported resolvase-like protein-encoding genes. The process probably involved sequence insertion and reversal, in which *rlgA* was truncated into two Δ*rlgA* genes and then orientated in opposite direction with the adjacent fragments **(**Figure 4c**)**. Spontaneous reversal of the *rlgA* gene is commonly observed in other virulence plasmids such as plasmid pST56-2, pST53-1, and pST90-2, even though they lacked the insertion fragment of ARI-B **(**Figure 4b**)**. In addition, pS1380-118kb-like plasmids only conferred phenotypic resistance to aminoglycoside antibiotics, since AMR genes including *aph(3’)-la*­, *aac(3’)-lld*, *dfrA12,* and *aadA2* were detected in the ARI-A region. BLASTN analysis showed that the ARI-A region from plasmid pS1380-118 kb exhibited homology (100% similarity, 68% ∼ 74% coverage) to plasmid pCTXM15_020026 (Accession number: CP034956), pECSE_01 (Accession number: KX683283) and pGMI17-003_3 (Accession number: CP031137), which were recovered from *E. coli* and lacked the genetic structure IS*26*-*mph(A)*-*mrx*-*mphR*-IS*6100*. This observation indicates that the macrolide-resistance encoding fragment might be derived from an exogenous plasmid, such as plasmid pS1380-135 kb.

## Discussion

non-typhoidal *Salmonella* is an important pathogen that causes food-borne diseases, the prevalence of which ranked second among a total of 31 pathogens that cause 9.4 million cases of food poisoning in the United State each year [30]. *Salmonella* is the etiologic agent of salmonellosis in humans causing severe diseases, especially in immunocompromised patients as well as children and the elderly [3]. Ciprofloxacin, ceftriaxone, and azithromycin are antibiotics commonly used for the treatment of salmonellosis. Upon decades of evolution and accumulation of multidrug resistance-encoding elements in the bacterial genomes, novel structures in multidrug resistance plasmids have emerged and were found to be responsible for causing a sharp increase in the rate of resistance to these three antibiotics in *Salmonella*. Our recent work demonstrated that the IncI1 plasmids could acquire various β-lactamase encoding determinants such as the *bla*_CTX-M_ group variants, resulting in an increasing prevalence of ceftriaxone resistance [31]. This type of plasmid was subjected to extensive studies as they may act as helper plasmids that mediate further acquisition of ciprofloxacin resistance-encoding genes by fusing with non-conjugative MDR plasmids or fragments containing such genes [32,33]. In addition, a recent report has recovered a conjugative IncI1 plasmid carrying the *erm(B)* and *bla*_CTX-M-104_ genes that encode resistance to both cefotaxime and azithromycin from a clinical *Klebsiella pneumoniae* strain [26]. These findings indicate that IncI1 type plasmids might play critical roles in the spreading of resistance genes and are worthy of further investigation. The emergence of novel ciprofloxacin resistance-encoding genetic elements has limited the usage of ciprofloxacin in the treatment of salmonellosis [34]; hence azithromycin is increasingly regarded as an important last-line antibiotic for the treatment of *Salmonella* infections.

To date, vast majority of *Salmonella enterica* serovar Typhimurium strains (88%) harbor a virulence plasmid of approximately 90 kb [35]. This virulence plasmid was reported to be non-conjugative, as it does not contain AMR genes and cannot be selected by antibiotics in conjugative assay [36]. However, Ahmer et al. confirmed that it was a self-transmissible plasmid in *Salmonella typhimurium*, exhibiting a transmission frequency of 2.9 × 10^−4^ transconjugants per donor [37]. Besides, virulence plasmids play significant roles in host adaptation and often affect bacterial vertical transmission among their preferred hosts [38]. Unlike virulence plasmids in other non-typhoidal *Salmonella* that are only detected in fecal samples and cause gastroenteritis, *S. typhimurium* and *S. enteritidis* containing virulence plasmids can recover from blood specimens and cause severe *Salmonella* bacteremia [39]. Furthermore, the absence of *Salmonella* plasmid virulence (*spv*) operons does not cause fatal infections in mice models, since virulence genes *spvBCD* are key factors that ruin host immunity [40]. In the past two decades, the genetic structures of these virulence plasmids have been extremely conserved, only several plasmids deposited in the NCBI database were found to have acquired aminoglycoside resistance genes.

In this study, we performed genetic characterization of two azithromycin-resistant *Salmonella* isolates that can successfully transfer their azithromycin resistance phenotype to *E. coli* J53 via filter mating experiments. We found that the azithromycin resistance phenotype of these two strains was encoded by IncI1 and IncFIC/IncFIB type conjugative plasmids that harbored the *erm(B)* gene, as well as the IS*26*-*mph(A)*-*mrx*-*mphR*-IS*6100* or IS*26*-*mph(A)*-*mrx*-*ΔmphR*-IS*26* cluster. We confirmed that these genetic elements could confer azithromycin-resistance, as *E. coli* DH5α strains which acquired the *erm(B)* gene [26] or the IS*26*-*mph(A)*-*mrx*-*mphR*-IS*6100* fragment exhibited an increase in MICs of azithromycin to over 128 fold, but those which acquired the *mph(A)* gene alone or the structure of IS*26*-*mph(A)*-*mrx*-Δ*mphR*-IS*26* did not. Importantly, we recovered a *Salmonella* virulence plasmid carrying the azithromycin resistance cluster IS*26*-*mph(A)*-*mrx*-*mphR*-IS*6100*; we hypothesized that it was the evolution product of azithromycin resistance plasmids, such as the plasmid pS1380-135 kb, upon extensive dissemination in *S. Typhimurium*.

In summary, this study identified two conjugative plasmids and a virulence plasmid that encoded resistance to azithromycin. Transmission of such azithromycin resistance-encoding plasmids in *Salmonella* not only limits the therapeutic options of *Salmonella* infections but also helps establish an AMR gene reservoir in *Salmonella*. The formation of novel multidrug resistance and virulence-encoding plasmids in *S. typhimurium* strains also leads to acceleration in the rate of dissemination of virulence plasmids as they would become more transmissible under the selection pressure of antimicrobial agents. Further surveillance and research on the transmission and evolution of virulence plasmids in *Salmonella* are necessary.

## Data Availability

The plasmids sequencing data of pS1330-110 kb, pS1380-118 kb, and pS1380-135 kb have been deposited into GenBank under the accession numbers OM048933, OM048934, and OM048935, respectively.
